# 1812. Impact of removing ESBL testing report on the selection of antibiotics for the treatment and 30-day mortality of patients infected with ESBL-producing organisms

**DOI:** 10.1093/ofid/ofac492.1442

**Published:** 2022-12-15

**Authors:** Sofia Waeuseng

**Affiliations:** Sungaikolok hospital, Thailand, mueng, Narathiwat, Thailand

## Abstract

**Background:**

The rising burden of carbapenem-resistant organisms is well recognized as a global crisis. Several studies have identified preceding carbapenem use as a risk factor for the subsequent development of infections with carbapenem-resistant, gram- negative organisms. We characterized the impact of removal of the ESBL designation from microbiology reports on inpatient antibiotic prescribing.

**Methods:**

A historical control study, interventional analysis study was conducted at Ramathibodi Hospital) to compared 1 year before removal ESBL designation (period1, August 1, 2019 to July 31, 2020) and 1 year post removal ESBL designation (period 2, August 1, 2020 to July 31, 2021) on inpatient antibiotic prescribing. Categorical variables were compared using the Fisher exact test. Continuous variables were evaluated using the Wilcoxon rank-sum test. All statistical tests were 2-tailed, and a *P < .05* was considered statistically significant.

**Results:**

The primary outcome definitive prescribing of carbapenems decreased from 56.5% to 41.3% afterward (*P*= .01). Prescription of cefepime was also decreased from 13.6% to 3.5% (*P*< .05), respectively. Whereas piperacillin-tazobactam use significantly increased from 10.4% to 28.7% (*P*< .05). Trimethoprim-sulfamethoxazole was prescribed more frequently, i.e., increased from 0 to 2.8%, but this change was not statistically significant. The secondary outcome 30-day mortality from any cause was no different between the two groups, with 22 of 154 patients (14.3%) in group 1 and 24 of 143 (16.8%) in group 2 (*P = .55*).

**Conclusion:**

Definitive prescribing of carbapenems was decreased and β-lactam–β-lactamase inhibitor combination was increased after removal ESBL report. Our findings confirm the importance of collaboration between microbiology and antimicrobial stewardship programs.

**Disclosures:**

**All Authors**: No reported disclosures.

